# 2000 Year-old Bogong moth (*Agrotis infusa*) Aboriginal food remains, Australia

**DOI:** 10.1038/s41598-020-79307-w

**Published:** 2020-12-17

**Authors:** Birgitta Stephenson, Bruno David, Joanna Fresløv, Lee J. Arnold, GunaiKurnai Land and Waters Aboriginal Corporation, Jean-Jacques Delannoy, Fiona Petchey, Chris Urwin, Vanessa N. L. Wong, Richard Fullagar, Helen Green, Jerome Mialanes, Matthew McDowell, Rachel Wood, John Hellstrom

**Affiliations:** 1 In the Groove Analysis Pty Ltd., Brisbane, QLD Australia; 2 Australian Research Council Centre of Excellence for Australian Biodiversity and Heritage, Canberra, ACT Australia; 3 Monash Indigenous Studies Centre, 20 Chancellors Walk, Monash University, Clayton, VIC 3800 Australia; 4 GunaiKurnai Land and Waters Aboriginal Corporation, Kalimna West, VIC Australia; 5 School of Physical Sciences, Environment Institute, and Institute for Photonics and Advanced Sensing (IPAS), University of Adelaide, Adelaide, SA Australia; 6 Laboratoire EDYTEM, Université Savoie Mont Blanc, 73376 Le Bourget du Lac Cedex, France; 7 Radiocarbon Dating Laboratory, University of Waikato, Hamilton, New Zealand; 8 School of Earth, Atmosphere and Environment, Monash University, Clayton, VIC Australia; 9 Centre for Archaeological Science, School of Earth, Atmospheric and Life Sciences, University of Wollongong, Wollongong, NSW Australia; 10 School of Earth Sciences, University of Melbourne, Parkville, VIC Australia; 11 School of Natural Sciences, University of Tasmania, Hobart, TAS Australia; 12 Radiocarbon Facility, Research School of Earth Sciences, Australian National University, Acton, ACT Australia

**Keywords:** Palaeoecology, Food webs

## Abstract

Insects form an important source of food for many people around the world, but little is known of the deep-time history of insect harvesting from the archaeological record. In Australia, early settler writings from the 1830s to mid-1800s reported congregations of Aboriginal groups from multiple clans and language groups taking advantage of the annual migration of Bogong moths (*Agrotis infusa*) in and near the Australian Alps, the continent’s highest mountain range. The moths were targeted as a food item for their large numbers and high fat contents. Within 30 years of initial colonial contact, however, the Bogong moth festivals had ceased until their recent revival. No reliable archaeological evidence of Bogong moth exploitation or processing has ever been discovered, signalling a major gap in the archaeological history of Aboriginal groups. Here we report on microscopic remains of ground and cooked Bogong moths on a recently excavated grindstone from Cloggs Cave, in the southern foothills of the Australian Alps. These findings represent the first conclusive archaeological evidence of insect foods in Australia, and, as far as we know, of their remains on stone artefacts in the world. They provide insights into the antiquity of important Aboriginal dietary practices that have until now remained archaeologically invisible.

## Introduction

Ethnographic accounts from around the world have reported the widespread use of insects as food by people^1–3^. In some cases, such as among the Shoshone and other Great Basin tribes of the U.S., swarms of grasshoppers and crickets were driven into pits and blankets^4^, while among the Paiute the larvae of Pandora moths (*Coloradia pandora lindseyi*) were smoked out of trees to fall into prepared trenches, where they would be cooked^5^. Across the world, insects could be mass-harvested, often seasonally, offering high nutritional value especially in fat, protein and vitamins^6^. The harvesting of insects in the past has ranged from opportunities to feed large communal gatherings during times of plenty, to more individualistic economic pursuits such as in the search for delicacies or the exploitation of low-ranked resources when other foods were scarce or depleted^7–9^. Irrespective of the catch, insects often represented an important component of the diet, and of the reliability and thus dependability of locales as resource zones, with implications for social scheduling and cultural practice. However, a paucity of archaeological studies of insect food remains has resulted in a downplay or omission of the use of insects from archaeological narratives and deep-time community histories^10^.

**Figure 1:**
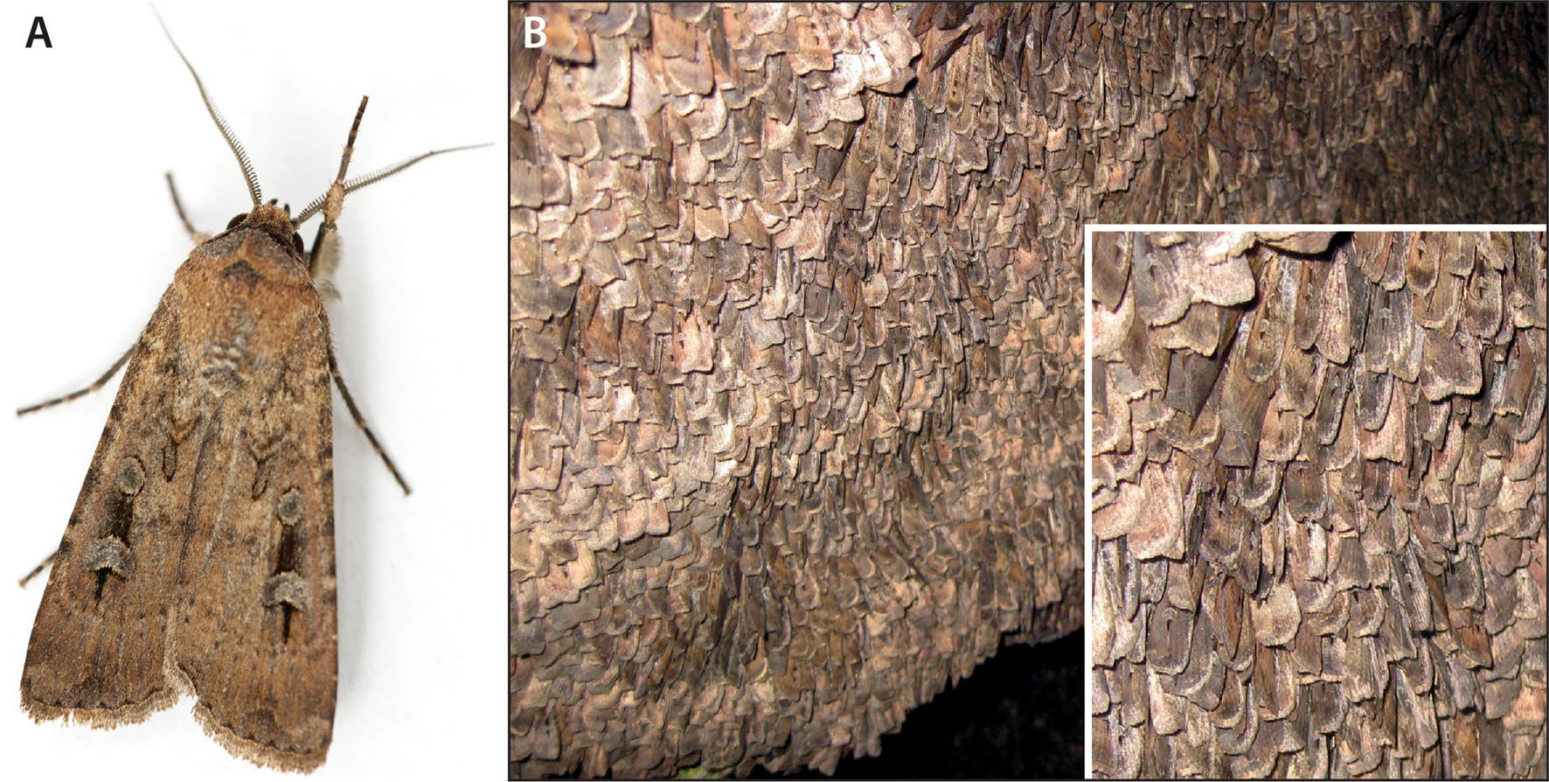
(**A**) Bogong moth, *Agrotis infusa* (photo: Ajay Narendra). (**B**) Thousands of moths per square metre aestivating on a rock surface (photo: Eric Warrant).

In Australia, a wide range of insects is known to have been eaten by Aboriginal groups, in particular the larvae (‘witchetty grubs’) of cossid moths (especially *Endoxyla leucomochla*) in arid and semi-arid areas^11–13^. Of particular interest to archaeologists and behavioural ecologists has been the seasonal consumption of Bogong moths by mass gatherings of Aboriginal groups in the southern portions of the Eastern Uplands^14^ (Fig. 1). However, no conclusive archaeological evidence has ever been reported for the processing or use of Bogong moths.

### The Cloggs Cave grindstone

**Figure 2:**
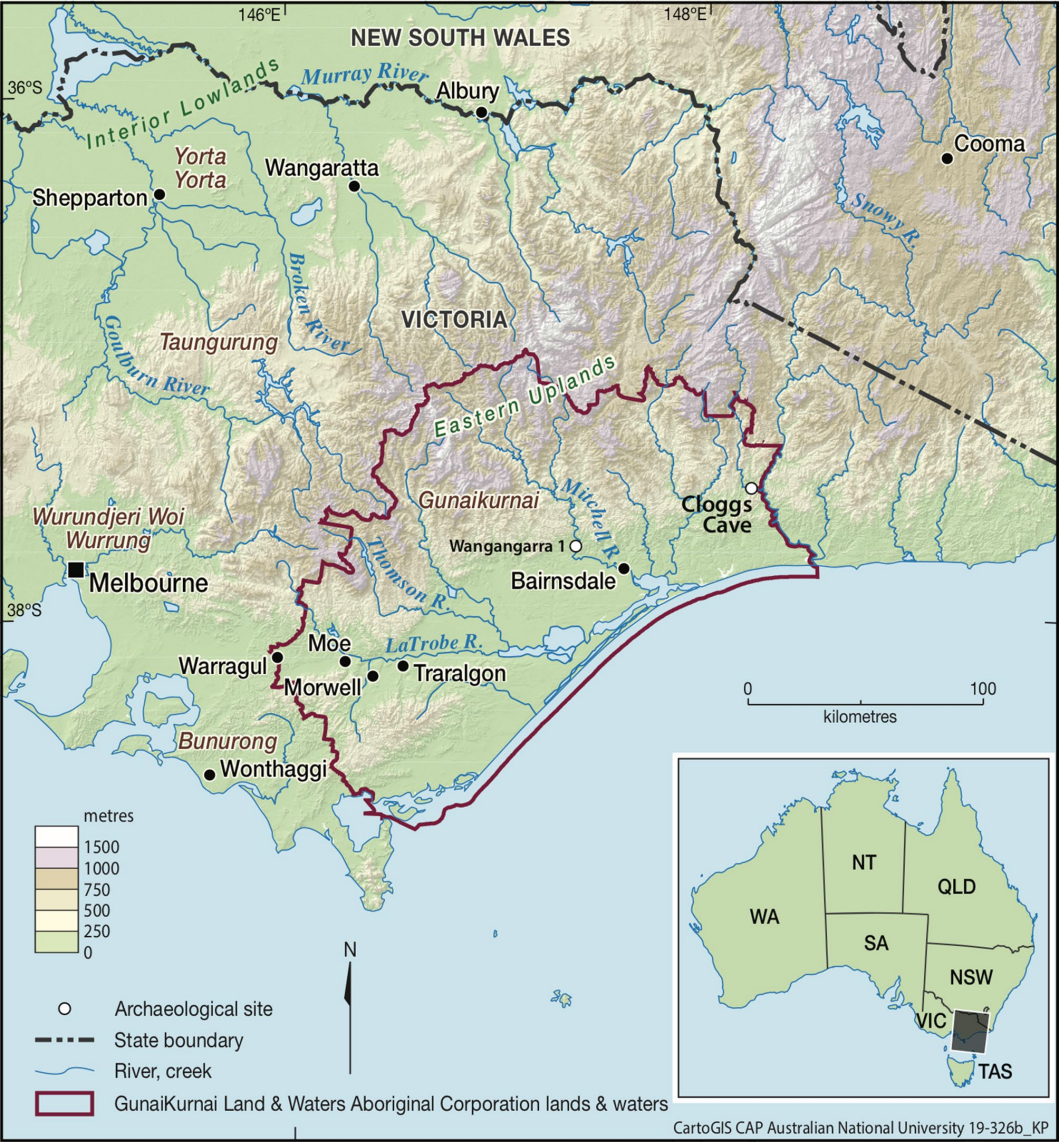
Location of Cloggs Cave and the area of the GunaiKurnai Land and Waters Aboriginal Corporation, at the southern foothills of the Australian Alps. Esri ArcMap 10.5 (https://desktop.arcgis.com/en/arcmap/) and Adobe Illustrator CC 2017 (21.0) (https://helpx.adobe.com/au/illustrator/release-note/illustrator-cc-2017-21-0-release-notes.html) were used by CartoGIS Services, College of Asia and the Pacific at the Australian National University, to create the map.

**Figure 3:**
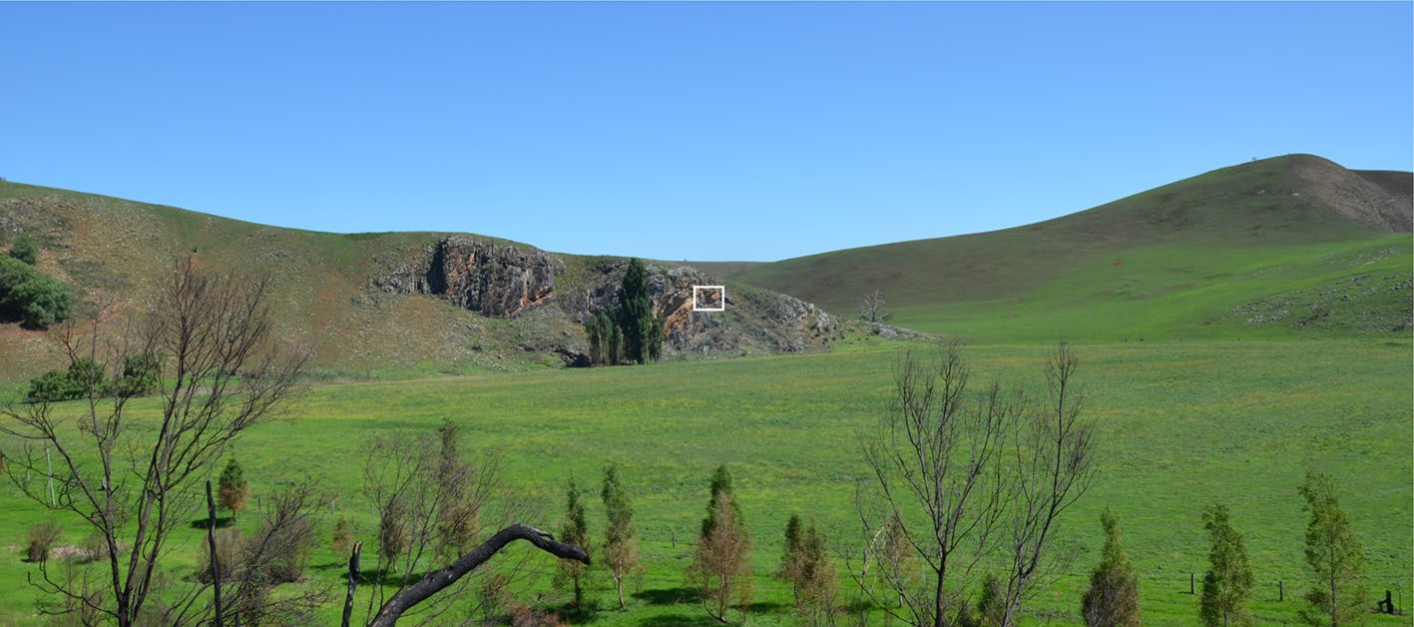
Cloggs Cave cliffline above the Buchan River flood plain, showing location of cave entrance (white rectangle) (photo: Bruno David).

Cloggs Cave is located 72 m above sea level in the southern foothills of the Australian Alps, in the lands of the Krauatungalung clan of the GunaiKurnai Aboriginal peoples of southeastern Australia (Fig. 2). The cave is a small, 12 m long × 5 m wide × 6.8 m high limestone karst formation that is today entered through a walk-through opening on the side of a cliff (Fig. 3). Indirect sunlight dimly illuminates the cave for much of the day (Supplementary Fig. S1).

Archaeological excavations were first undertaken in 1971–1972^14^, followed by a new program of excavations in 2019–2020, initiated by the GunaiKurnai Land and Waters Aboriginal Corporation and directed by Bruno David. The new excavations were aimed at better determining the site’s stratigraphy and the antiquity of Aboriginal occupation (Supplementary Fig. S2). An intensive dating programme showed that the oldest excavated evidence for human activity dates to between 19,330–19,730 cal BP (median age of 19,530 cal BP; cal BP = before AD1950) and 20,590–23,530 cal BP (median age of 21,690 cal BP) (all calibrated radiocarbon ages in the text are presented at 95.4% probability range. See “Methods”; Supplementary Fig. S3)^15–17^.

During the 2019 excavations, a small, flat grindstone was found. The finely stratified hearth layers of stratigraphic unit (SU) 2 in which it was found were radiocarbon-dated to 1567–1696 cal BP at their top (uncalibrated: 1724 ± 16 BP; median age of 1632 cal BP) and 2002–2117 cal BP at their base (uncalibrated: 2091 ± 16 BP; median age of 2062 cal BP). The grindstone therefore dates to between 1600 and 2100 years ago (see “Methods”; Supplementary Figs. S3 and S4)^17^. No other grindstone has been found at Cloggs Cave.

**Figure 4:**
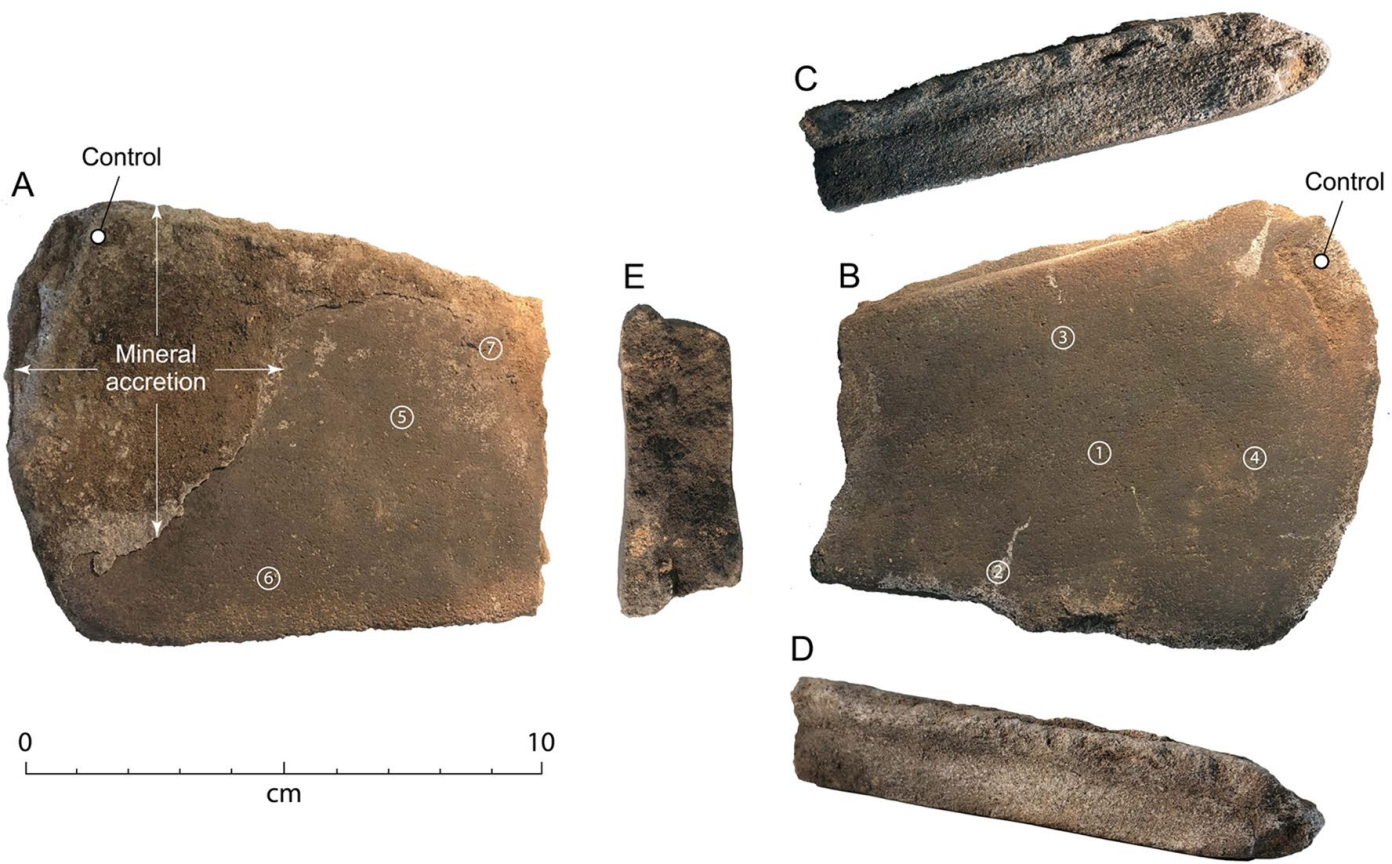
The Cloggs Cave grindstone. (**A**) Surface A, with the accretion that formed across parts of the surface after its use. (**B**) Surface B. (**C**) Margin A. (**D**) Margin B. (**E**) Narrow end. The numbers in circles are the residue sample numbers; the ‘control’ samples are in areas where grinding did not take place (photos: Richard Fullagar).

The grindstone is a tabular fragment of sandstone with two flat and parallel ground surfaces (Surfaces A and B), in the form of a flat dish (Fig. 4). It measures 10.5 cm long × 8.3 cm wide × 2.2 cm thick and weighs 304 g. The outer, intact margin is elliptical in plan view; the other three margins indicate old breaks that have been subsequently worn from use. Therefore, prior to its deposition at Cloggs Cave, the grindstone had been used in its current form.

To understand how the grindstone was used, we undertook use-wear and residue analyses (see “Methods”). The central area of both its surfaces contain fine unidirectional striations (Supplementary Figs. S5A and S5B), a lowered but not levelled topography, and areas of missing or ripped quartz grains (Supplementary Figs. S5C and S5D). Its use to shape ground stone axes is an unlikely function because the Cloggs Cave grindstone surfaces are relatively flat with only very slight concavities, and the lowered surface topography (Fig. 4) lacks broad grooves typical of axe grinding.

When viewed at lower (up to 5 ×) magnification under a stereozoom microscope with a point source of light, each surface appears relatively rough compared with grindstones used for processing seeds, which, in Australia, tend to be highly smoothed and polished^18,19^. There are numerous ‘pits’ where sand grains have been plucked from the surface during use (Supplementary Fig. S5D). The presence of a lowered surface topography (Supplementary Fig. S5C) with a lack of smooth, developed polish suggests that the stone was not used to process siliceous plants.

The repeated mechanical action of grinding has been shown to force residues into the voids and interstitial spaces of ground surfaces, where they become trapped^20–22^. Residue analyses conducted on grindstones worldwide have relied on microscopic observations of individual residue morphologies. However, visually diagnostic features can be altered by the mechanical forces of grinding, heat, and contact with water and various environmental factors, which can cause residues to swell or become amorphous^21–24^. The distinctiveness of residue identifications can be enhanced significantly with the introduction of biochemical staining that can be observed under high-power microscopy and is best used in conjunction with microscopic use-wear analysis and identification of residue morphologies^22^.

We extracted nine samples, or ‘lifts’, for residue analysis from across Surface A and Surface B of the Cloggs Cave grindstone, including a control sample from an unworked part of each surface (Fig. 4; see “Methods”). These samples were analysed using a recently developed biochemical staining technique that enables residues to be identified from colorimetric changes occurring at a cellular level, rather than relying solely on structural features (see “Methods”)^22^. We used the collagen stain Picrosirius Red (PSR) to differentiate between plant and animal residues (see “Methods”). When PSR comes into contact with collagen (a protein unique to animals), it reacts to produce clear and distinctive staining and enhanced birefringence in cross-polarised light^22,25^.

### Residues extracted from the grindstone

A range of residues were identified in the lifts, including amorphous collagen, collagen fibres, collagen structures, partially woven collagen, possible bone-like fragments, moth wing segments, a possible moth hind leg, amorphous cellulose, wood-like structures with pits, carbonised material, bordered pits and minerals (see below).

We found collagenous residues in mid-range densities across Samples 1 and 4 from Surface B and across Sample 5 from Surface A (Supplementary Fig. S6). These extractions were taken from central areas across each modified surface. In all cases, the frequency of the collagenous residues was approximately three times greater than the collagenous residues associated with the control samples. Residues include damaged collagen fibres of varying thicknesses, including some reticular fibres.

Woven collagen structures clearly show birefringence in cross-polarised light across Sample 1. Woven collagen, which forms quickly, is mechanically weak and usually associated with immature bone. Although woven collagen may persist as tendon and ligament attachments to bone, it is generally replaced by organised parallel collagen fibre bundles at skeleton maturity^26^. Collagen fibrils are found in the connective tissues of vertebrates as well as in invertebrates such as insects^27^, and may be present as individual strands, woven structures or parallel bundles, including among the Lepidoptera (moths and butterflies)^28^.

The density and combination of collagenous residues on the Cloggs Cave grindstone indicates that it was used to process fauna. A variety of collagenous materials (including woven collagen) were found in association with carbonised residues across Sample 2, which was extracted from a crystalline layer. The residues present on Samples 1 and 2 suggest that an insect or immature vertebrate was prepared and cooked using the grindstone.

We identified a moderate density of carbonised plant residues across Sample 2, in particular, wood-like structures with pits. These ranged from being partially to completely carbonised. Partially carbonised residues were also seen across Sample 4. In addition, bordered pits in small clusters were identified, along with pits within the carbonised structures. Bordered pits are cavities that are essential components in the water-transport system of higher-order plants and are found in the lignified cell walls of xylem conduits (vessels and tracheids). The pit membrane allows water to pass between xylem conduits, but limits the spread of embolism and vascular pathogens in the xylem^29^. Small quantities of lignin were also present (see “Methods”). Lignin is found in the cell walls of vascular plants (especially in wood and bark) and is responsible for the rigidity of plant structures.

The residues identified via biochemical staining are consistent with the use of twigs and bark as fuel for fires such as those of the microstratified ashy layers in which the grindstone was found (see Supplementary Fig. S3)^17^. Partially carbonised wood-like material was also noted across Sample 5. The density and distribution of carbonised residues varies across extractions. Our observations suggest either that: (a) the stone has been placed in or near fires; or (b) ash, embers or fires of varying heat were placed or lit across the stone, for varied durations of time.

We identified especially high densities (frequency of residue particles per unit volume of sample) of amorphous cellulose across Samples 1, 2, 4 and 5 (Supplementary Fig. S7). The presence of partially carbonised amorphous cellulose indicates that the plant residues were associated with fire. While the high density is indicative of a plant-processing event, there is no evidence of combinations of plant residues normally expected from plant processing. In particular, no starch grain or phytolith was seen in any of the extractions. While low heat can damage starch and cause its structure to be disrupted and its characteristic extinction-cross to be lost, low heat does not completely destroy starch visibility^30^. Similarly, phytoliths can be reshaped but not destroyed by fire^31^. The presence of animal and mineral residues but absence of starches and phytoliths is thus interpreted as a true absence of plant processing activities rather than a taphonomic effect of environmental factors negatively impacting their preservation.

**Figure 5:**
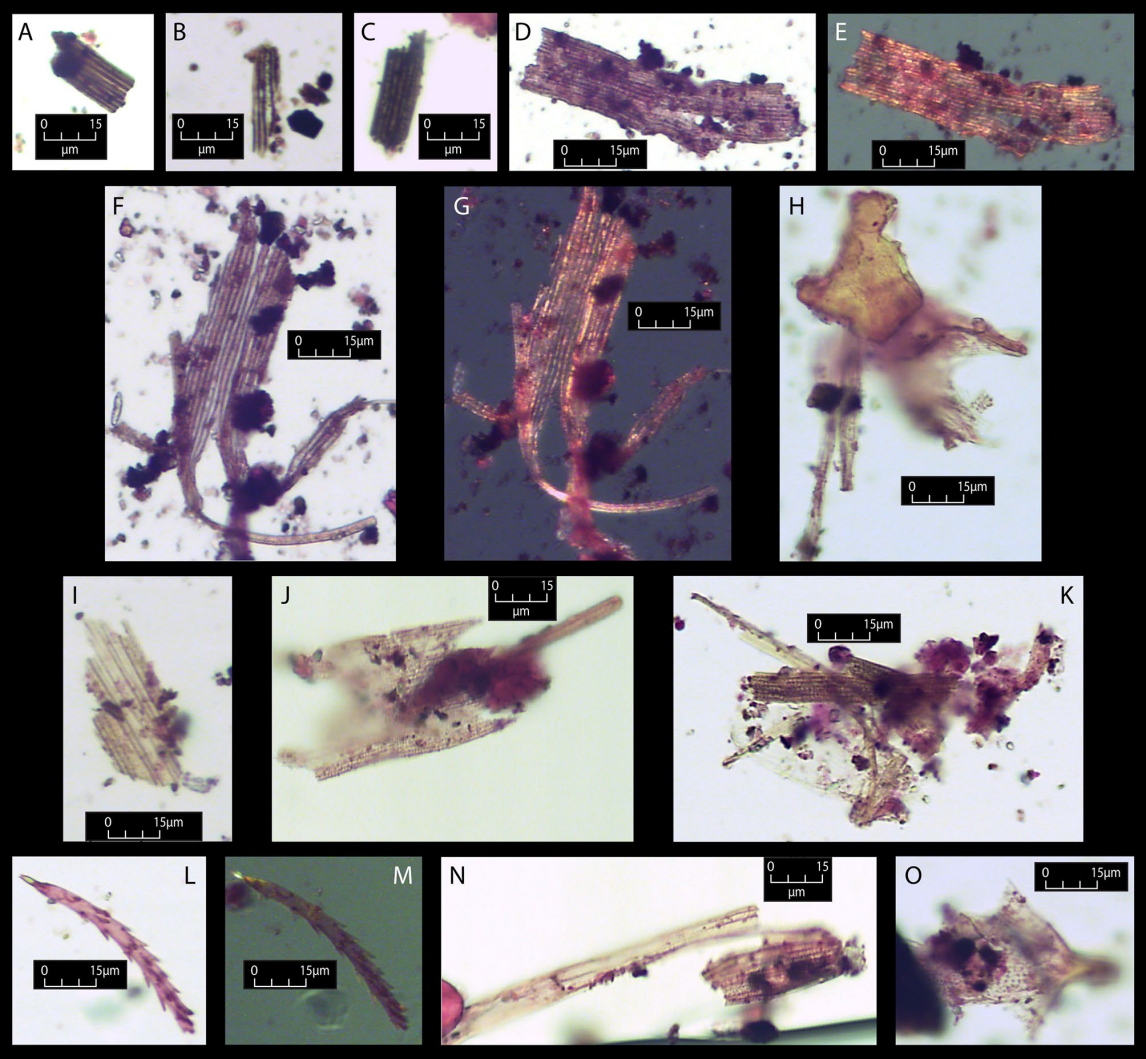
Examples of Bogong moth segments from lifted samples (all at × 400 magnification). (**A**) Partially carbonised wing structures from Sample 2 (pp). (**B**) Partially carbonised wing structure and carbonised material from Sample 2 (pp). (**C**) Partially carbonised moth wing segment from Sample 4 (pp). (**D–E**) Damaged moth wing segment from Sample 6 (**D** pp; **E** xp). (**F–G**) Damaged moth wing segment from Sample 6 (**F** pp; **G** xp). (**H**) Damaged moth wing segment with proteinaceous material, from Sample 6 (pp). (**I**) Unburnt moth wing segment from Sample 4 (pp). (**J**) Damaged moth wing segment with attachment, from Sample 6 (pp). (**K**) Damaged moth wing segments from Sample 6 (pp). (**L–M**) Probable moth hind leg from Sample 6 (**L** pp; **M** xp). (**N**) Damaged moth wing segment from Sample 6 (pp). (**O**) Damaged moth wing segment with attachment, from Sample 6 (pp). Light source = plane (pp), part polarised (part pol) and cross-polarised (xp) (photos: Birgitta Stephenson).

We found a high density of variably carbonised insect wings in Sample 6 (Surface A), and lower densities in Samples 2 and 4. These wing fragments contain regular patterning or structure and exhibit distinct birefringence in cross-polarised light. A portion of proteinaceous material was associated with a ‘tangle’ of these structures (Fig. 5). To assess whether the insect remains were those of the Bogong moth, we compared the residues on Samples 2, 4 and 6 with a comparative reference sample (see “Methods”). All 26 cases of wing segments from the grindstone matched the metrical and morphological characteristics of those from Bogong moths in the reference material. The recorded damage on the archaeological wing segments, such as ripped wing structures, small rectangular wing fragments and tearing in various states of carbonisation, is what would be expected from ethnohistoric accounts of Bogong moth processing. Aboriginal people from across the region are known to have cooked Bogong moths on heated earth during the early and mid-nineteenth century. The moths were stirred during cooking, causing the wings and legs to be broken off by friction and heat. Some of the moths were pounded and ground into a paste which could then be smoked to preserve the food for weeks^1,2^.

## Discussion: implications for the deep-time history of Bogong moths as an Aboriginal food

At the time of early contact with European explorers and settlers in the late 1820s and early 1830s, Aboriginal peoples of southeastern Australia harvested Bogong moths during their summer movements into the high mountains^32^. Each spring (September), Bogong moths migrate south over 1000 km from warmer climes. Travelling at night, the moths’ journeys last many days, arriving in the Australian Alps where, over the summer months (late September–March), they lie dormant (aestivate) in the hundreds of thousands among the protected rocky outcrops^3,11,14^.

Every Aboriginal group from the plains and foothills surrounding the alpine region had estates extending into the high peaks. The moth was said by Aboriginal informants to be highly nutritious, easily harvested and very palatable. In the northern area of the Bogong Peaks, south of Tumut, New South Wales, Aboriginal people stupefied the moths with smoke and cooked them in a fire from which the coals were removed^1^. If desired, the moths could then be placed on a sheet of bark or in a wooden bowl and ground with stick or pebble to make a cake^1,2^. These cakes could be smoked to preserve them for several weeks. The only conventional archaeological evidence likely to remain from these cultural practices are the pebbles, but such stones were also used for a wide range of other purposes, such as to rub the insides of animal pelts in the making of fur coats for warmth, as heat retainers in earth ovens, or as hammerstones for the manufacture of stone tools. Smooth pebbles have previously been found in several open sites above the tree-line in the Mount Kosciusko region of New South Wales and at lower altitudes. Based on their distribution in high altitude camps, their size and shape, and, in two archaeological cases, white fluorescence under ultra-violet light, it was concluded that they were associated with the pounding and grinding of Bogong moths, hitherto the only purported known examples of Bogong moth processing tools. The fluorescence was assumed to have been caused by proteins from the oil of moth abdomens^14^. However, many other organic materials fluoresce white under ultraviolet light, including chlorophyll; carotenoids from plants, bacteria, algae and fungi; cellulose; minerals such as calcite, gypsum and quartz; bone; wax; and oil from human handling, rendering these early interpretations highly equivocal and thus unreliable^33,34^. The Cloggs Cave findings reported here represent the first demonstrable archaeological evidence of Bogong moth processing as described in historical accounts. The results of our study indicate that Aboriginal peoples of southeastern Australia were harvesting, preparing and cooking Bogong moths for food 1600–2000 years ago, allowing for the scheduling of the associated summer feasts going back at least 65 generations.

The cessation of the annual Bogong moth festivals within three decades of colonial intrusion in and surrounding the Australian Alps^32^ until their revival in the twentieth century, coupled with what has been until now an inability to recover definitive archaeological traces of Bogong moths, has denied their inclusion in deep-time Aboriginal histories. The Cloggs Cave findings now show that Bogong moths were processed and eaten for at least 1600–2000 years. They also demonstrate a viable means to investigate where and when Bogong moths were used prior to the colonial period of ethnohistory.

## Methods

### Excavation methods

Squares P34 and P35, each 50 × 50 cm in area, were excavated from the cleaned southeast wall of the 1972 main pit, which had remained opened for 47 years. The excavation proceeded to a maximum 2.28 m depth, without reaching bedrock. Excavation proceeded in arbitrary excavation units (XUs) of typically < 2 cm (and at times < 1 cm) thickness following the stratigraphic layers (SUs) in which they lay.

The cave and its surroundings were mapped in three dimensions using LiDAR, the sediments were geomorphologically investigated, and the Squares P34–P35 and associated southeastern wall stratigraphic sequence was dated through a combination of 40 accelerator mass spectrometry (AMS) radiocarbon, eight optically stimulated luminescence (OSL) and nine uranium-series (U-series) ages. Two additional OSL samples were dated from the lowermost strata of the northeastern wall of the 1971–1972 pit. The details of these excavation methods have been published elsewhere^16^.

The small grindstone reported here came from a c. 15 × 10 × 5 cm block of sediment that collapsed from the upper part of the recently cleaned southeast wall of the 1971–1972 pit during the 2019 excavation (see Supplementary Fig. S4). The block of sediment was securely matched to the specific micro-stratified hearth layers from which it fell. The grindstone was embedded within the collapsed block. It lay flat and parallel to the individual ashy microlayers.

### The sediment sequence

The layers of Squares P34 and P35 are broadly similar to those identified in 1971–1972. We identified a c. 1.8 m-deep deposit of poorly differentiated and, at times, rocky layers (SU3A–3G) superimposed by a complex set of 73 finely stratified and mostly ash-rich layers (SU2A–SU2BU), itself topped by two continuous thin layers (also incorporating a minor lens) of brown (dry Munsell: 7.5YR 4/2–10YR 4/3) sandy loam (SU1A–SU1C) (Supplementary Fig. S4). The SU3 layers represent a subsidence crater that rapidly filled with sediments eroding from upslope. The overlying set of finely stratified SU2 layers consist mainly of well-differentiated and inter-merging thin layers of ash from camp fires, some of which have sudden boundaries. The SU2 layers are in situ, having gradually accumulated over the infilled SU3 subsidence crater. The grindstone came from this set of finely stratified SU2 ash layers.

Squares P34–P35 were dated from the base of SU3G to near the top of the finely stratified SU2 ash layers (Supplementary Fig. S4). The entire c. 1.7 m from the base of the excavation to the SU3A–SU3B interface built up rapidly c. 6090 ± 1140 years ago, immediately after the formation of the subsidence crater, as determined by the radiocarbon, OSL and U-series ages^15^. This rapid build-up of 1.7 m of sediment was followed by slower sedimentation of SU3A and of the lowermost layers of SU2 until c. 4400 cal BP. The fine layers of ash then began 2091 ± 16 BP (2002–2117 cal BP), ceasing 1724 ± 16 BP (1567–1696 cal BP). A single fire was built more recently, 142 ± 25 BP (6–281 cal BP)^15,17^.

### The excavation chronology

Full details of the radiocarbon, OSL and U-series ages, and associated dating methodologies for the Cloggs Cave sequence, have been published elsewhere^15–17^. Here we provide a synthesis of the OSL ages obtained from the northeastern and southeastern wall sequences, as well as the radiocarbon ages obtained from Squares P34–P35 and the associated southeastern wall sequence.

### AMS radiocarbon ages

Samples dated at the Accelerator Mass Spectrometry ^14^C facility, University of Waikato (laboratory code Wk-), were prepared as follows. Charcoal, leaf, and possum scat samples were pretreated using 1 M HCl at 80 °C, followed with multiple hot 1 M NaOH washes and then reacidified with 1 M HCl at 80 °C. Each sample was then rinsed to neutral using Milli-Q water and dried at 80 °C. The supernatant was removed by pipette after each step. NaOH treatments were repeated until no colour remained on application of NaOH to the sample. CO_2_ was collected by oxidation at 800 °C overnight in the presence of pre-baked CuO and silver wire. The cryogenically separated CO_2_ was then reduced to graphite with H_2_ at 550 °C using an iron catalyst. Pressed graphite was analysed at the Keck Radiocarbon Dating Laboratory, University of California^35^. Over the period of measurement, replicate measurements of known age standards were used for background correction and quality control (‘Ancient kauri charcoal’ blank [av. F^14^C = 0.0012 ± 0.0003 (gSD); n = 9]; ‘Oak charcoal’ [av. F^14^C = 0.6186; 3858 ± 11 BP (gSD); n = 8] and ‘Lake Rotomahana charcoal’ (av. F^14^C = 0.2120; 12,462 ± 16 BP; n = 2]. All ^14^C results were fractionation-corrected using on-line AMS δ^13^C values. These are not reported. Percent carbon (%C) on combustion was used to assess for charcoal quality. Wood charcoal is expected to yield carbon percentages of > 50%. Where charcoal is poorly preserved (low %C yield), it is possible that the chosen pretreatment technique may not have effectively removed the contamination.

At the Australian National University (laboratory code S-ANU#), charcoal was cleaned with a scalpel under a low-power binocular microscope, crushed to around 1 mm, and subjected to an acid (1 M HCl, 70 °C, 30 min), base (1 M NaOH, 70 °C, 1 h, changed until solution remained colourless) and acid (1 M HCl, 70 °C, 30 min) pre-treatment. After each step, the charcoal was washed three times in Milli-Q water, or until the solution remained colourless. Freeze-dried charcoal was combusted in a sealed quartz tube in the presence of CuO wire and Ag foil at 900 °C. CO_2_ was cryogenically purified and collected prior to graphitisation with H_2_ over an Fe catalyst. Graphite was dated in a Single Stage NEC AMS at the ANU^36^, and dates calculated using an AMS-derived δ^13^C. Replicate measurements of known age standards were used for background correction and quality control. %C was estimated volumetrically during cryogenic collection^37^.

Samples Wk-48860 and S-ANU 60824 were measured as part of a blind test and came from the same piece of charcoal and produced statistically different results within one sigma (χ^2^_1:0.05_ = 9.03 < 3.84). However, the age determinations are within three sigma. It is uncertain whether the difference is a statistical issue or something more. The scale of the offset does not affect our interpretations.

### Single-grain OSL ages

OSL measurements were made using the experimental apparatus, single-aliquot regenerative-dose (SAR) procedures, and quality assurance criteria described previously^38,39^, and further detailed elsewhere^16^. OSL dating samples were collected from cleaned exposure faces using metal or opaque PVC tubes, and were immediately sealed with light-proof plastic upon extraction (Supplementary Figs. S3A and S3B). Purified quartz grains with a diameter of 212–250 μm were prepared for equivalent dose (D_e_) analysis under safe light conditions (dim red LEDs) at the University of Adelaide. Between 800 and 1100 single-grain D_e_ measurements were made for each sample using aluminium discs drilled with a 10 × 10 array of 300 μm-diameter holes. All of the OSL samples exhibit scattered single-grain D_e_ distributions that are consistent with syn-depositional mixing with pre-existing cave deposits prior to burial^40^ (Supplementary Figs. S3C and S3D). Consequently, we have used the minimum age model^41^ to derive representative burial dose estimates from the well-bleached grain populations that were derived directly from the cave exterior prior to burial (Supplementary Table S2).

Dose rate evaluations have been undertaken using a combination of in situ gamma-ray spectrometry^42^ and low-level beta counting^43^, taking into account cosmic ray contributions^44^, an assumed minor internal alpha dose rate^45^, beta-dose attenuation^46,47^ and long-term water content^48,49^, as detailed in Supplementary Table S2.

### Usewear

The grindstone was initially examined under reflected light microscopes (Olympus BH2 metallographic microscope at 100 ×, 200 × and 500 × magnifications; and Nikon SMZ1B stereozoom microscope at 10 × –50 × magnifications) by one of our team members (RF). A second team member (BS) then examined the grindstone under a Dino-Lite portable microscope. Use-wear (including striations, surface levelling, plucked quartz grains and differential smoothing/polish on upper and lower topography) were noted and compared with experimental grindstones documented on Australian sandstones of varying degrees of hardness^50^ and other ground stone tool experiments undertaken by others^51^.

Fine, unidirectional striations are visible on the central area of Surface A. They radiate from under a large area of mineral accretion and run parallel to Surface A’s long margins (Fig. 4; Supplementary Figs. S5A and S5B). The irregular margin and location of the striations indicate that the accretion became affixed to the surface after the grinding had taken place.

The topography of the fine striations across the central area of Surface A varies and has not been levelled. Irregular pit-like features occur within the matrix. The remaining grains are intact, and spaces or pits are due to the ripping of non-modified natural grains. The slightly higher surface adjacent to Margin B displays fine striations. The high points of matrix grains are lowered but not levelled, and a low or poorly developed polish is present (Supplementary Figs. S5C and S5D). Matrix grains between the central area of Surface A and Margin B are rounded rather than levelled, and display an irregular, undulating topography.

### Residue sampling

In total, nine residue samples were extracted from the Cloggs Cave grindstone (see Fig. 4). Samples 1 and 4 came from the central area of Surface B; Sample 2 was lifted from an area of the mineral crystalline deposit along Margin B of Surface B; and Sample 3 was extracted from an area near Margin A of Surface B. A control sample was taken from a non-ground area of Surface B where a small piece of the sandstone became detached after the final grinding event (Fig. 4). Four samples were extracted from Surface A, including Sample 5 from the central area with striations, Sample 6 from the slightly higher topography associated with Margin B, and Sample 7 from the broken area toward Margin A. A control sample was lifted from an area adjacent to the accretion on Surface A (Fig. 4).

Residue sampling of the Cloggs Cave grindstone was undertaken with the aid of a Dino-Lite portable microscope. A 60 μl drop of ultra-purified water was placed on an area of interest using a volumetric pipette and left to soak for 5–10 min depending on the porosity of the sample area. A second drop of ultra-purified water was placed on the same area and the pipette used to agitate the soaked matrix. A second pipette with water was then inserted to suction material from the soaked matrix. Sampled areas were transferred to slides using a standard wet-mount procedure. The slides were covered with pre-cleaned Petri dish lids and dried under lights. Using established protocols^22^, the dried slides were stained with Picrosirius Red for enhanced visibility under the microscope (see below).

After biochemical staining of the residues, collagen was identified under cross-polarised light. The larger collagen fibres appear bright orange or yellow, and thinner fibres including reticular fibres appear green. The microscopic properties of starch and other plant material are not altered by PSR staining. We tested for the presence of lignin by staining samples with acidified Phloroglucinol (Wiesner reagent). The Wiesner reagent is used to identify lignin by turning lignified tissue a characteristic cherry red or red violet^52^.

Weathering can distort or remove residues, and poor preservation conditions can markedly bias the quantity and type preserved^52^. It is thus important to examine the residue distribution and relationships to working margins and associated use-wear to help determine whether a residue is use-related. Control samples are essential to help assess whether a residue is likely to be use-related. In this study, extractions taken from adjacent non-worked surfaces served as control samples^53,54^.

High-powered microscopy (200 ×–400 ×) using a Leitz Dialux 22 microscope with polarising capability was used to examine the stained slides. A Tucsen ISH 500 camera was used to photograph lifted residues in plane, part polarised and cross-polarised light at 250 × and 400 × magnifications.

A comparative reference sample of dried Bogong moth remains, including wings and hind legs, was pounded across a glass slide. The slide was then stained with PSR following the developed protocol (see above). The slide was examined under high-powered microscopy and images were used as comparative references (Supplementary Fig. S8). The comparative reference sample included long rectangular portions of wing, oval wing sections with attachment, torn/damaged wing segments and leg (Supplementary Fig. S8). The morphology and distinctive staining and birefringence of the reference Bogong moth wings in cross-polarised light was identical to the wing-like structures highlighted across Sample 6 (e.g. Fig. 5D–G).

## Supplementary Information

Supplementary Information.

## Data Availability

The datasets generated during and/or analyzed during the current study are available from the corresponding author on reasonable request.
