# Supplementary material for: 2000 Year-old Bogong moth (Agrotis infusa) Aboriginal food remains, Australia
Source: Sci Rep. 2020 Dec 17;10:22151. doi: 10.1038/s41598-020-79307-w (PMC7747710; doi:10.1038/s41598-020-79307-w)
Supplement: Supplementary file 1 — Supplementary Information. [file 41598_2020_79307_MOESM1_ESM.pdf]

Supplementary information

2000 year-old Bogong moth (*Agrotis infusa*) Aboriginal food remains, Australia

Birgitta Stephenson, In the Groove Analysis Pty Ltd., Brisbane, QLD (Australia). itg.analysis@gmail.com

Bruno David*, Monash Indigenous Studies Centre, Monash University, Clayton, VIC; Australian Research Council Centre of Excellence for Australian Biodiversity and Heritage (Australia). bruno.david@monash.edu

Joanna Fresløv, Kalimna West, VIC (Australia). jfreslov@glawac.com.au

Lee J. Arnold, School of Physical Sciences, Environment Institute, and Institute for Photonics and Advanced Sensing (IPAS), University of Adelaide, Adelaide SA; Australian Research Council Centre of Excellence for Australian Biodiversity and Heritage (Australia). lee.arnold@adelaide.edu.au

GunaiKurnai Land and Waters Aboriginal Corporation, Kalimna West, VIC (Australia). russell.mullett@glawac.com.au

Jean-Jacques Delannoy, Laboratoire EDYTEM, Université Savoie Mont Blanc, F-73376 Le Bourget du Lac Cedex (France); Australian Research Council Centre of Excellence for Australian Biodiversity and Heritage (Australia). jean-jacques.delannoy@univ-smb.fr

Fiona Petchey, Radiocarbon Dating Laboratory, University of Waikato, Hamilton (New Zealand); Australian Research Council Centre of Excellence for Australian Biodiversity and Heritage (Australia). fiona.petchey@waikato.ac.nz

Chris Urwin, Monash Indigenous Studies Centre, Monash University, Clayton, VIC; Australian Research Council Centre of Excellence for Australian Biodiversity and Heritage (Australia). chris.urwin@monash.edu

Vanessa N. L. Wong, School of Earth, Atmosphere and Environment, Monash University, Clayton, VIC (Australia). vanessa.wong@monash.edu

Richard Fullagar, Centre for Archaeological Science, School of Earth, Atmospheric and Life Sciences, University of Wollongong, NSW (Australia). fullagar@uow.edu.au

Helen Green, School of Earth Sciences, University of Melbourne, Parkville, VIC; Australian Research Council Centre of Excellence for Australian Biodiversity and Heritage (Australia). helen.green@unimelb.edu.au

Jerome Mialanes, Monash Indigenous Studies Centre, Monash University, Clayton, VIC; Australian Research Council Centre of Excellence for Australian Biodiversity and Heritage (Australia). jerome.mialanes@monash.edu

Matthew McDowell, School of Natural Sciences, University of Tasmania, Hobart, TAS; Australian Research Council Centre of Excellence for Australian Biodiversity and Heritage (Australia). matthew.mcdowell@utas.edu.au

Rachel Wood, Radiocarbon Facility, Research School of Earth Sciences, Australian National University, Acton, ACT; Australian Research Council Centre of Excellence for Australian Biodiversity and Heritage (Australia). rachel.wood@anu.edu.au

John Hellstrom, School of Earth Sciences, University of Melbourne, Parkville, VIC (Australia). j.hellstrom@unimelb.edu.au

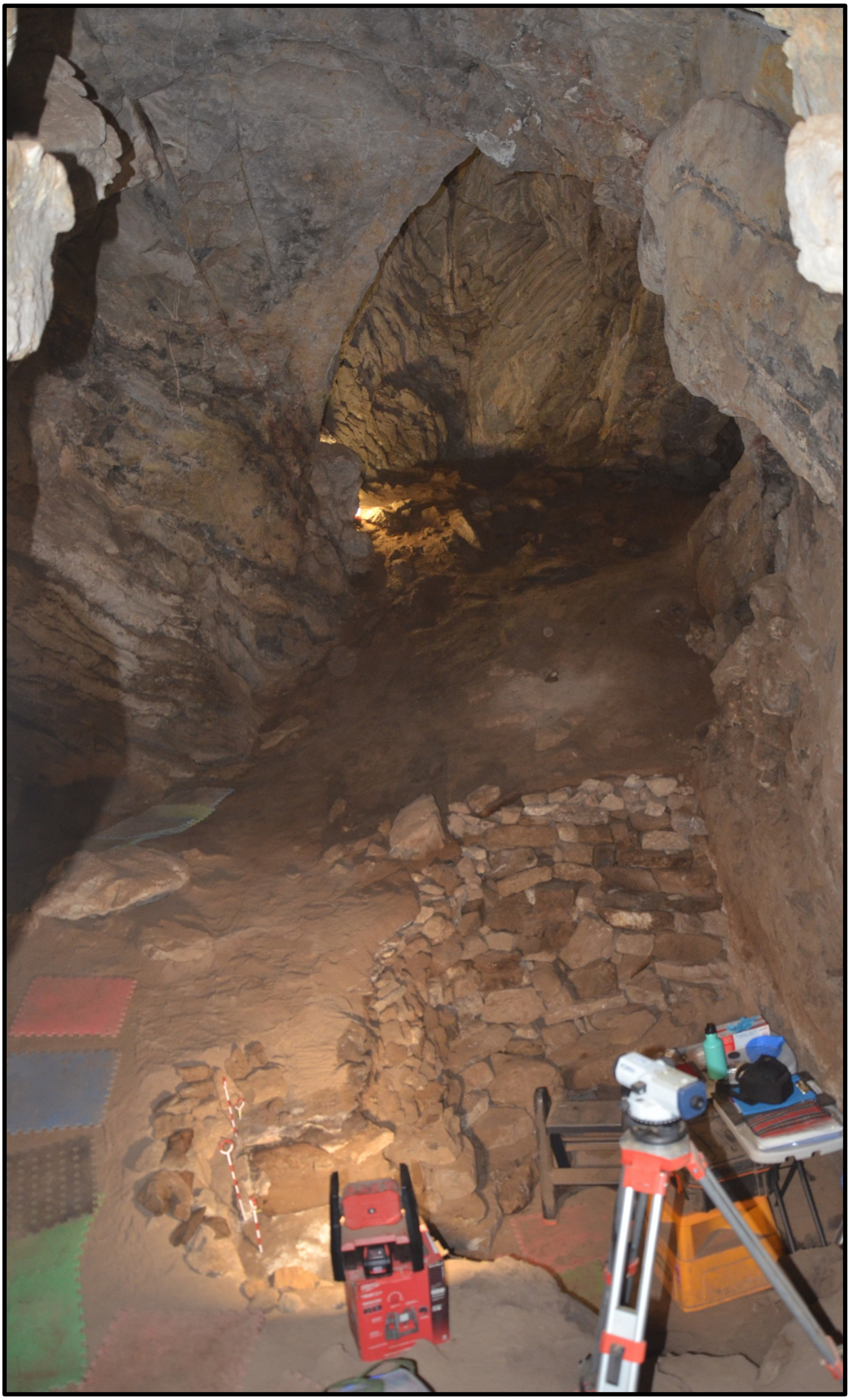

Supplementary Fig. S1. Inside Cloggs Cave looking south, 2020 excavations of Square R31 in progress. The walls of the 1971–1972 pit have been protected with a dry-stone wall (photo: Bruno David).

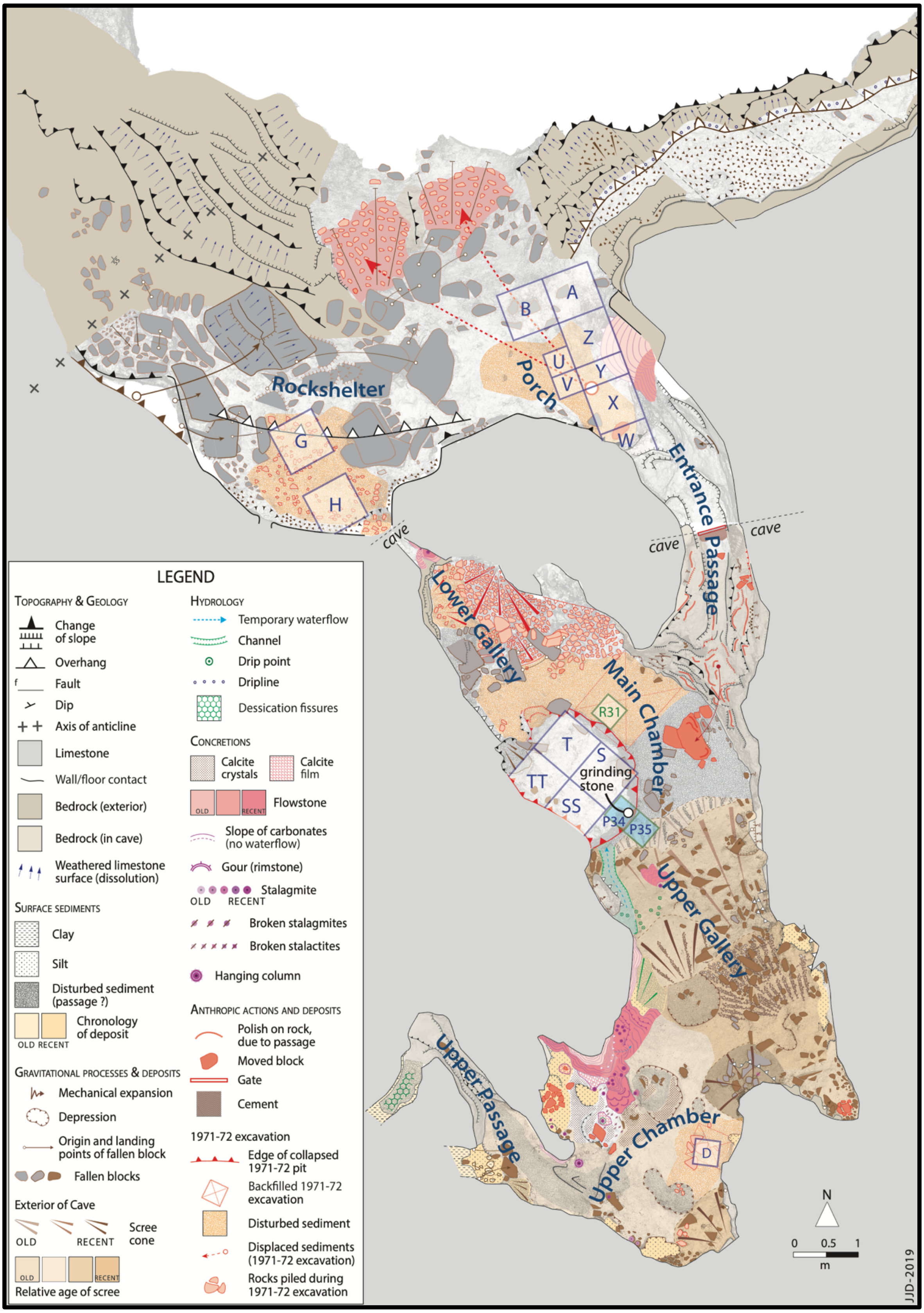

Supplementary Fig. S2. Map of the Cloggs Cave ground floor showing the zones of the site, excavation squares and the grindstone find-spot. The map was created through detailed geomorphological field annotations on a plan-view print of a purpose-made LiDAR scan and digitised in Adobe Illustrator CC 2017 (21.0) (https://helpx.adobe.com/au/illustrator/release-note/illustrator-cc-2017-21-0-release-notes.html) (image: Jean-Jacques Delannoy with modifications by Bruno David).

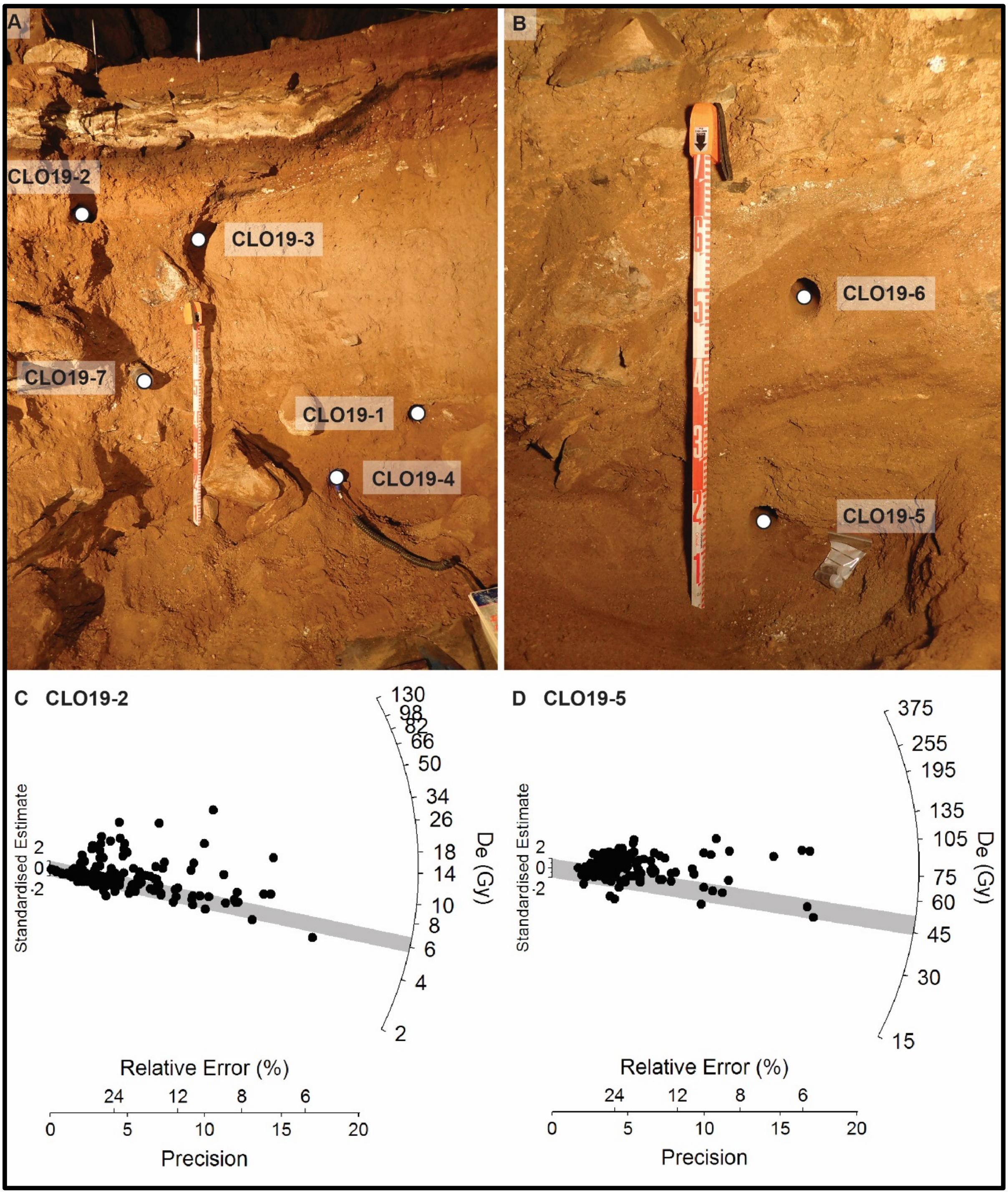

Supplementary Fig. S3. (**A**–**B**) Photos of selected OSL sample positions on the cleaned southeastern (A) and northeastern (B) walls of the 1971–1972 pit. (**C**–**D**) Examples of single-grain OSL equivalent dose (D_e) distributions for OSL samples CLO19-2 and CLO19-5, shown as radial plots. The grey bands are centred on the D_e values used for the age calculations, which were derived using the 3-parameter minimum age model for these two samples (see Supplementary Table S2) (photos and radial plots: Lee J. Arnold).

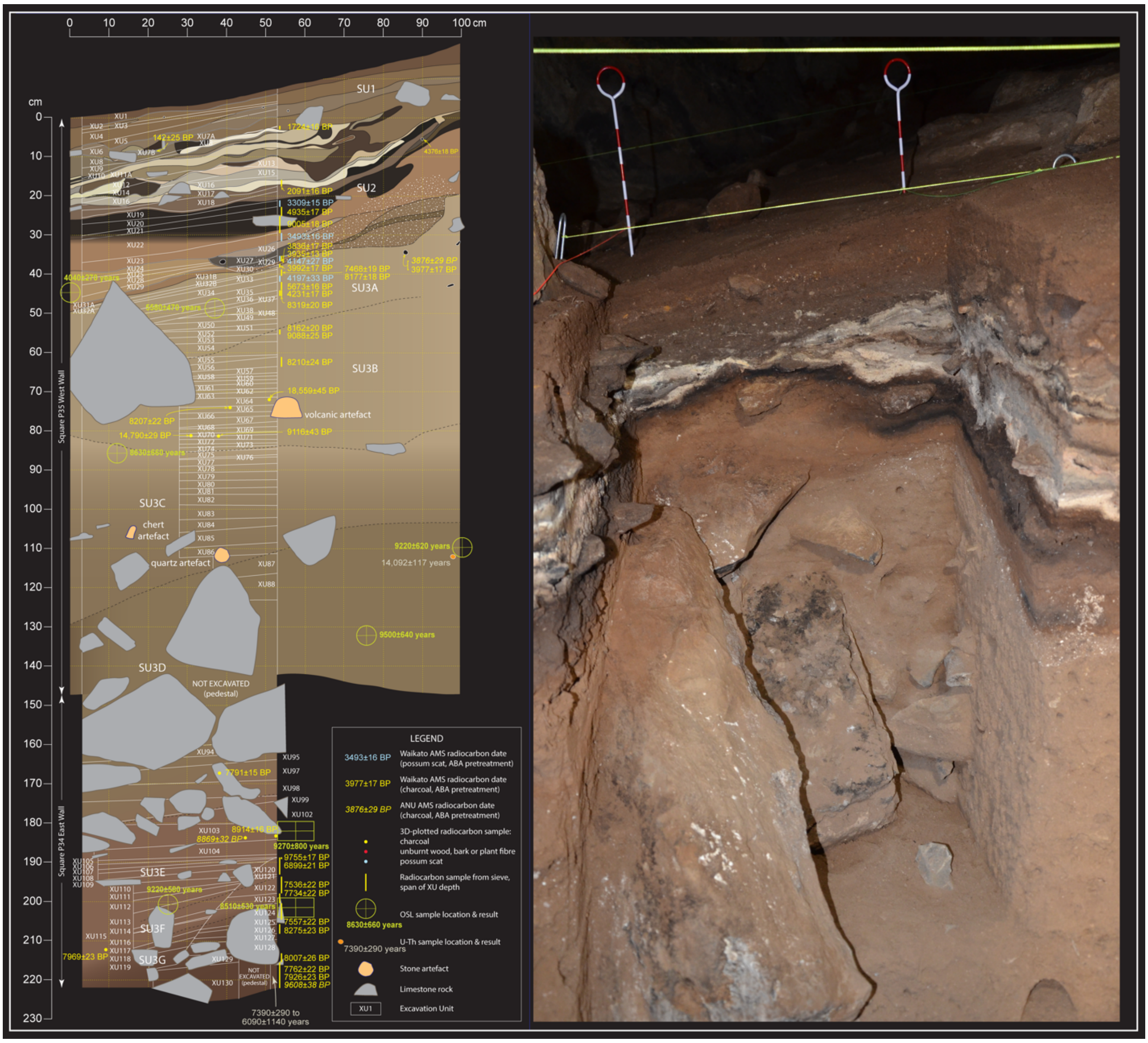

Supplementary Fig. S4. Stratigraphic details of Squares P34 and P35, Cloggs Cave. **Left**, Section drawing of Squares P34 and P35 with plotted radiocarbon ages. The section was drawn from the southeast wall of the 1971–1972 pit. Note that while SU1 and SU2 are well stratified in situ sediments, SU3 consists of redeposited sediments in an infilled subsidence cavity. **Right**, Square P35 excavation in progress, showing the fine ash layers from which the grindstone came, superimposed on the sediments of the infilled subsidence crater. The upper yellow stringline is set horizontal. The section drawing was drafted on graph paper in the field through a combination of tape-measure readings from a horizontal stringline and automatic level readings relative to a fixed datum point (both at 1 mm precision). The depth of the XUs and samples for the radiocarbon ages were back-plotted from automatic level readings. The figure was drawn in Adobe Illustrator CC 2017 (21.0) (https://helpx.adobe.com/au/illustrator/release-note/illustrator-cc-2017-21-0-release-notes.html) (section drawing and photo: Bruno David).

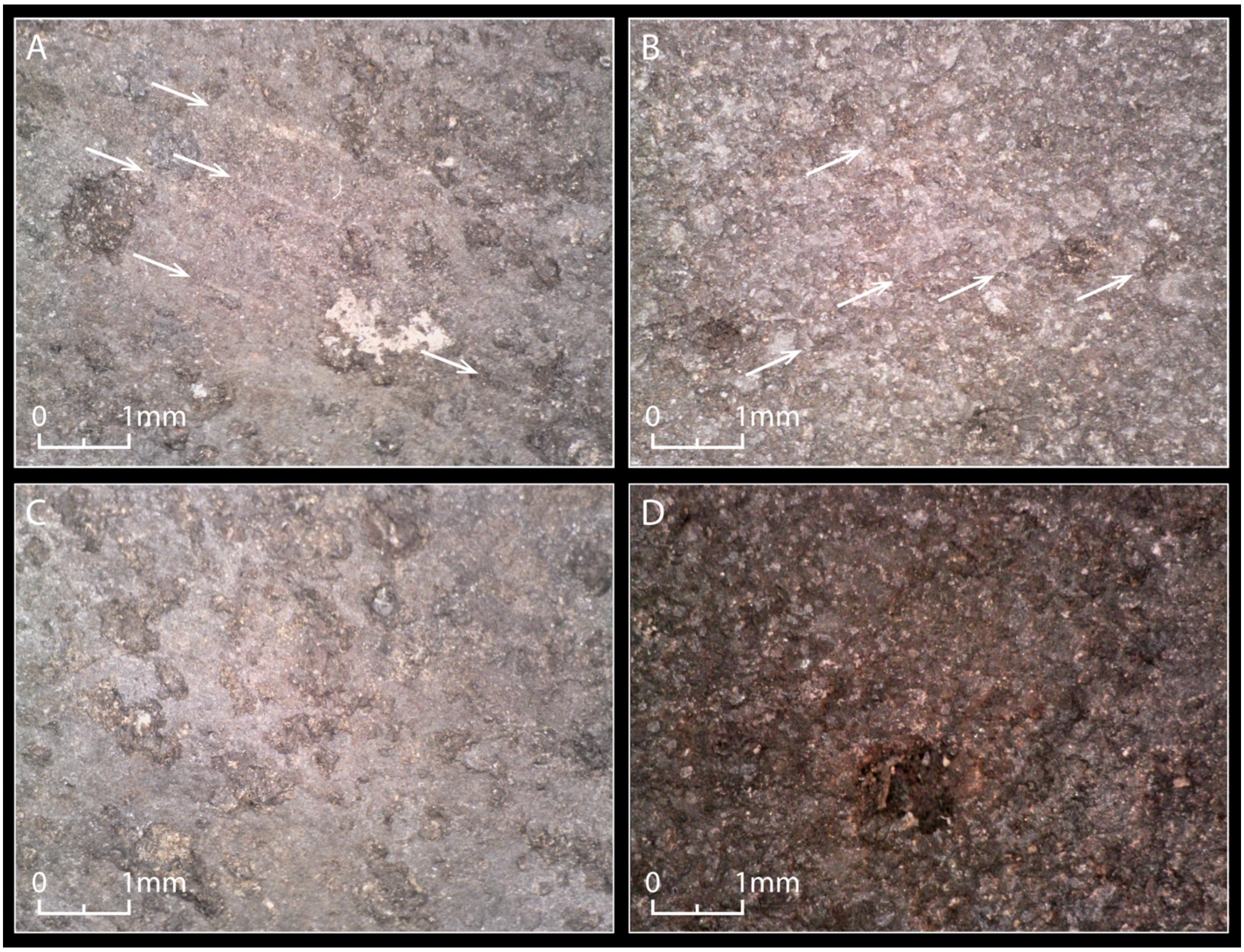

Supplementary Fig. S5. Surface topography of the Cloggs Cave grindstone ('magnification' refers to the microscope lens at which an image was taken). (**A**) Fine striations (identified by the white arrows) across the middle of Surface A (70× magnification). (**B**) Fine striations (identified by the white arrows) on Surface B (64× magnification). (**C**) Lowered surface topography of Surface A, between centre and Margin B (76× magnification). (**D**) Ripped matrix grains on Surface B (110× magnification) (photos: Birgitta Stephenson).

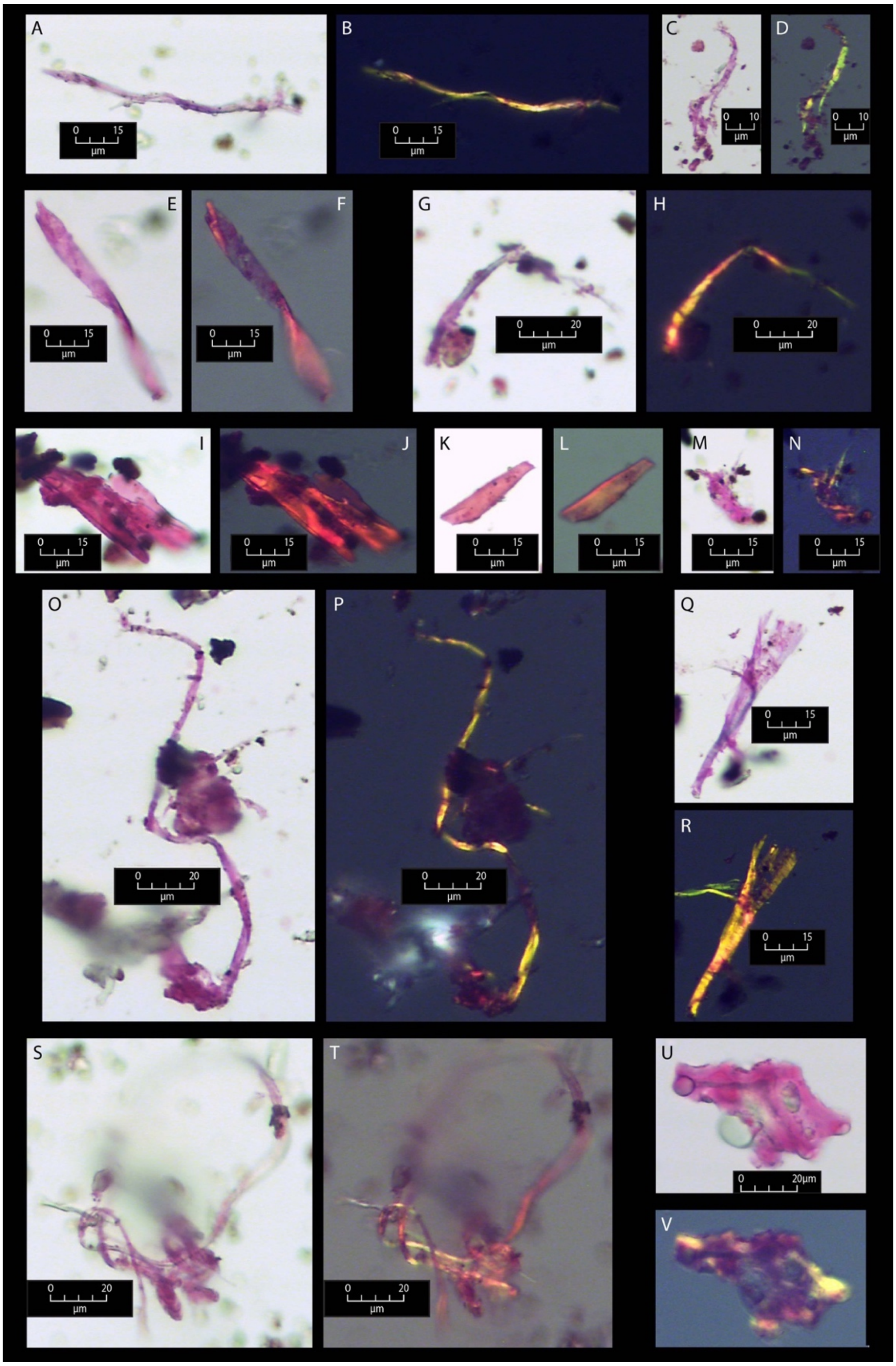

Supplementary Fig. S6. Examples of collagenous materials from lifted samples (all at 400× magnification). (**A–B**) Damaged collagen fibre from Sample 1 (A: pp; B: xp). (**C–D**) Collagen fibres from Sample 5 (C: pp; D: xp). (**E–F**) Damaged collagen fibre from Sample 1 (E: pp; F: xp). (**G–H**) Collagen fibre from Sample 4 (G: pp; H: xp). (**I–J**) Collagenous structure and partially carbonised material from Sample 2 (I: pp; J: xp). (**K–L**) Collagenous structure from Sample 2 (K: pp; L: xp). (**M–N**) Partially woven collagenous structure from Sample 1 (M: pp; N: xp). (**O–P**) Collagen fibre from Sample 4 (O: part polarised; P: xp). (**Q–R**) Unravelling collagen fibres from Sample 5 (Q: pp; R: xp). (**S–T**) Twisted collagen fibres from Sample 4 (S: part polarised; T: xp). (**U–V**) Woven collagen from Sample 2 (U: pp; V: xp) (photos: Birgitta Stephenson).

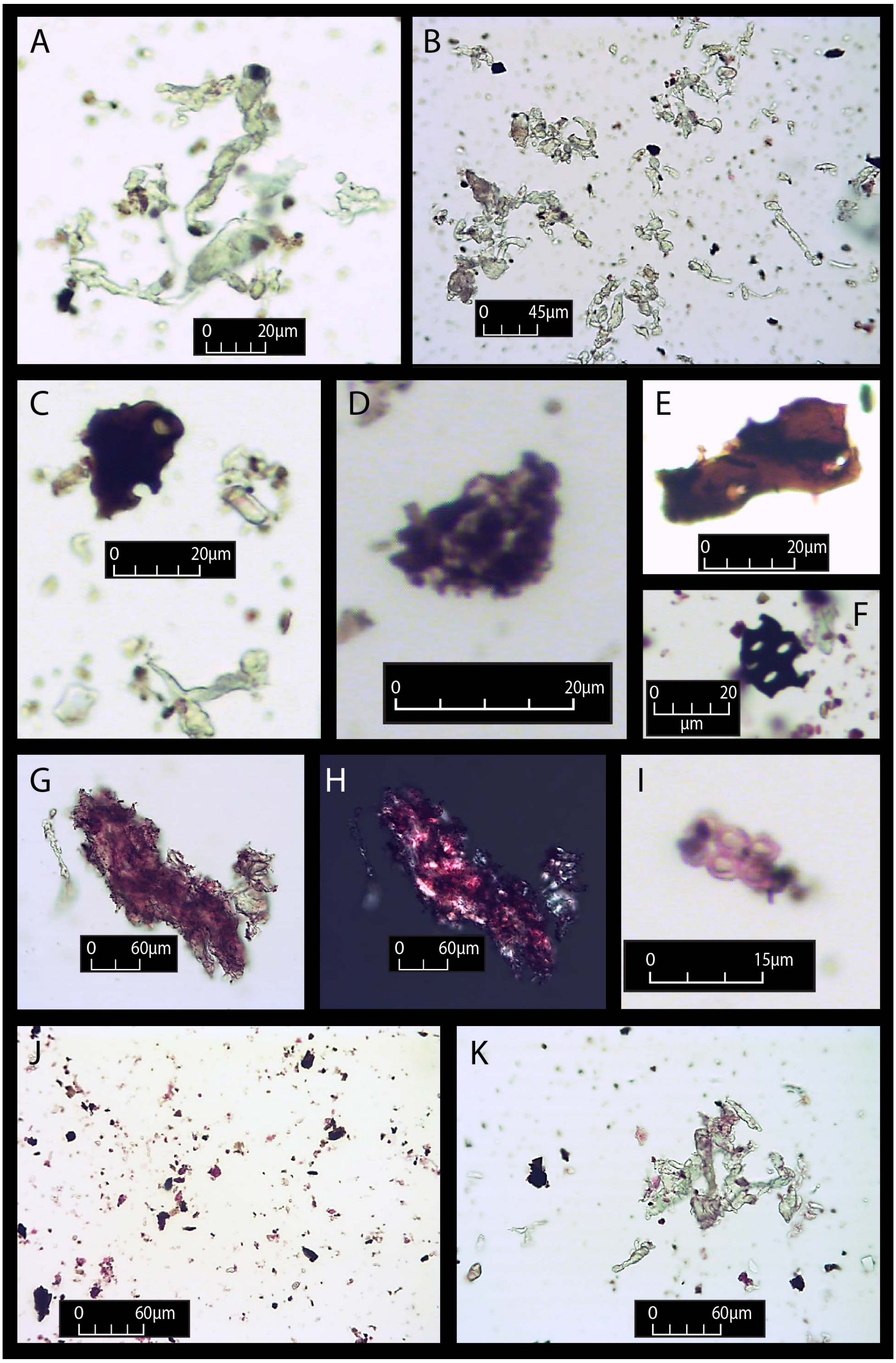

Supplementary Fig. S7. Examples of plant residues from lifted samples (all at 400× magnification). (**A**) Amorphous cellulose from Sample 1 (pp). (**B**) High-density amorphous cellulose from Sample 1 (pp). (**C**) Amorphous cellulose and carbonised plant-like material with pits from Sample 1 (pp). (**D**) Carbonised amorphous cellulose from Sample 1 (pp). (**E**) Partially carbonised material from Sample 5 (pp). (**F**) Carbonised wood-like material with pits from Sample 6 (pp). (**G–H**) Amorphous cellulose from Sample 3 (G: pp; H: xp). (**I**) Bordered pits from Sample 1 (pp). (**J**) Moderate density carbonised material from Sample 4 (part polarised). (**K**) Amorphous cellulose and carbonised material from Sample 5 (pp) (photos: Birgitta Stephenson).

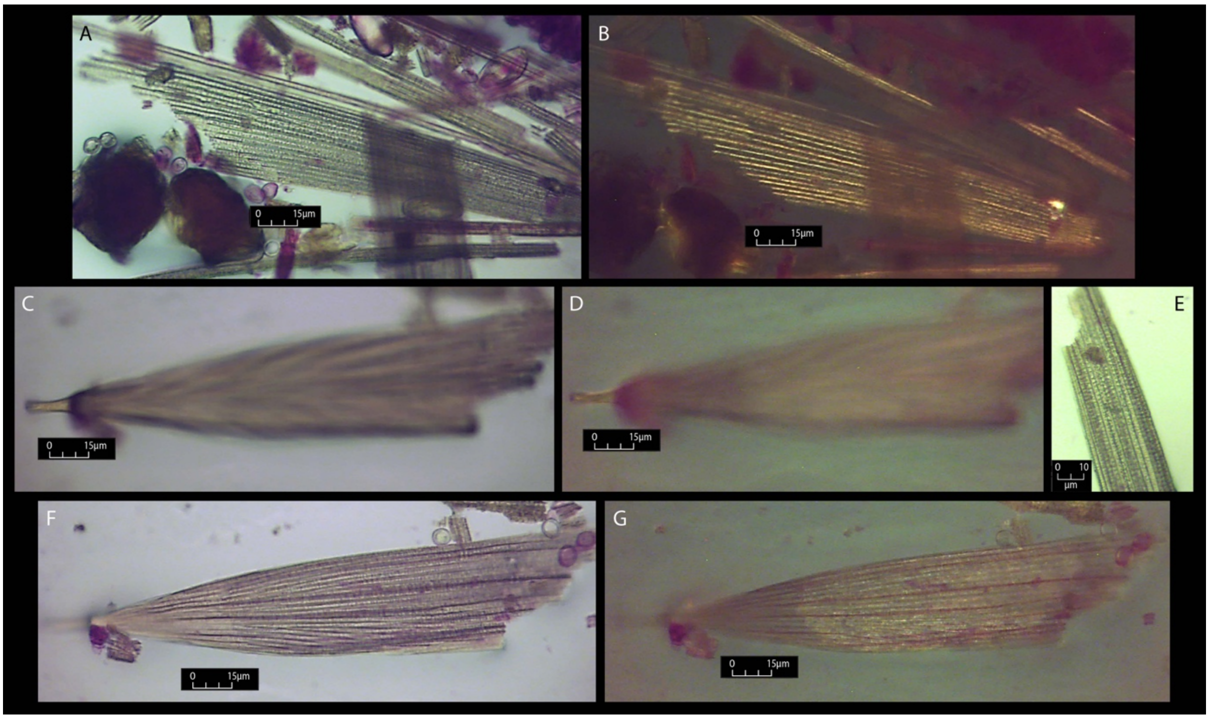

Supplementary Fig. S8. Comparative reference material of pounded dried Bogong moths (all at 400× magnification). (**A–B**) Segments of wing and other parts (A: pp; B: xp). (**C–D**) Attached section of wing (C: pp; D: xp). (**E**) Wing segment (pp). (**F–G**) Attached section of wing, focused on form (F: pp; G: xp) (photos: Birgitta Stephenson).

Supplementary Table S1. AMS radiocarbon ages on single pieces from Squares P34 and P35, Cloggs Cave. [a] Collected from the wall of the cleaned exposed 1971–1972 pit (and plotted on the section drawing) prior to commencement of the 2019 excavation (i.e., this sample does not have an XU attribution).

SU	XU	Square	Material Dated	Laboratory Code	δ^{13}C (‰)	^{14}C Age (BP)	%C
2E	7B	P35	*Eucalyptus* leaf	Wk-49638	n/a	142 ± 25	56.2
2N-2O-2T	8	P35	charcoal	Wk-49639	-29.1 ± 0.2	1724 ± 16	55.7
2AZ-2BB-2BH	17	P35	charcoal	Wk-49641	-22.4 ± 0.2	2091 ± 16	72.5
2BL	20	P35	possum scat	Wk-50442	n/a	3309 ± 15	n/a
2BL-2BP	21	P35	charcoal	Wk-49645	n/a	4935 ± 17	63.4
2BL-2BP	22	P35	charcoal	Wk-49646	-24.6 ± 0.2	9005 ± 18	73.5
2BL-2BP	23	P35	possum scat	Wk-50443	-24.5 ± 0.6	3493 ± 16	n/a
2BP-2BT	24	P35	charcoal	Wk-49648	-26.7 ± 0.2	3836 ± 17	63.0
2BS-2BU	29	P35	charcoal	Wk-49502	-25.7 ± 0.3	3935 ± 13	78.1
2BT	28	P35	charcoal	Wk-49650	-25.0 ± 0.2	3992 ± 17	64.5
2BS-2BU	29	P35	possum scat	Wk-50444	n/a	4147 ± 27	n/a
2BI	SE wall[a]		charcoal	Wk-48865	n/a	4376 ± 18	38.6
2BX-3A	SE wall[a]		charcoal	S-ANU 60824	n/a	3876 ± 29	56
				Wk-48860	n/a	3977 ± 17	56.9
3A	33	P35	charcoal	Wk-49503	-27.5 ± 0.3	7468 ± 19	71.5
3A	33	P35	charcoal	Wk-49504	-25.3 ± 0.4	8177 ± 18	73.7
3A	34	P35	possum scat	Wk-50445	n/a	4197 ± 33	n/a
3A	35	P35	charcoal	Wk-49652	-24.3 ± 0.2	5673 ± 16	65.1
3A	36	P35	charcoal	Wk-49648	-22.6 ± 0.2	4231 ± 17	63.0
3A	46	P35	charcoal	Wk-49108	n/a	8319 ± 20	63.4
3A-3B	52	P35	charcoal	Wk-49109	-27.0 ± 0.5	8162 ± 20	66.6
3A-3B	52	P35	charcoal	Wk-49110	-24.5 ± 0.5	9088 ± 25	68.1
3B	56	P35	charcoal	Wk-49111	-25.4 ± 0.5	8210 ± 24	63.4
3B	64	P35	charcoal	Wk-49114	-23.0 ± 0.5	18,559 ± 45	66.0
3B	65	P35	charcoal	Wk-49115	n/a	8207 ± 22	59.9
3B	71	P35	charcoal	Wk-49116	-24.8 ± 0.5	14,790 ± 29	69.5
3B	72	P35	charcoal	Wk-49118	n/a	9116 ± 43	46.9
3D	97	P34	charcoal	Wk-49211	-26.7 ± 0.6	7791 ± 15	68.4
3E	103 B	P34	charcoal	Wk-49212	-25.4 ± 0.6	8914 ± 16	65.6
3E	103 B	P34	charcoal	S-ANU 61804	n/a	8869 ± 32	55
3E	106	P34	charcoal	Wk-49505	-25.4 ± 0.3	9755 ± 17	70.6
3E	120	P34	charcoal	Wk-49364	-24.9 ± 0.6	6899 ± 21	67.7
3E-3F	122	P34	charcoal	Wk-49365	-25.9 ± 0.6	7536 ± 22	68.2
3F	123	P34	charcoal	Wk-49366	-25.0 ± 0.6	7734 ± 22	73.9
3F-3G	124	P34	charcoal	Wk-49367	-25.6 ± 0.6	7557 ± 22	58.1
3G	126	P34	charcoal	Wk-49368	n/a	8275 ± 23	72.4
3G	118	P34	charcoal	Wk-49327	-23.4 ± 0.5	7969 ± 23	64.4
3G	129	P34	charcoal	Wk-49369	-23.1 ± 0.5	8007 ± 26	70.6
3G	130	P34	charcoal	Wk-49370	-23.0 ± 0.5	7762 ± 22	70.4
3G	130	P34	charcoal	Wk-49371	-23.9 ± 0.5	7926 ± 23	71.4
3G	130	P34	charcoal	S-ANU 62431	n/a	9608 ± 38	57

Supplementary Table S2. Single-grain OSL age summary for the Cloggs Cave samples*. Equivalent dose (D_e) determination was undertaken on individual quartz grains with diameters of 212–250 µm. The total dose rate includes an internal dose rate component of 0.03 Gy/ka, with an assigned relative uncertainty of ±30%. The total uncertainty on the final OSL age represents the 1σ range, and includes a systematic component of ±2% associated with laboratory beta-source calibration.

Sample name	Unit	Water content [a]	Environmental dose rate (Gy/ka)				Equivalent dose (D_e) data			OSL age (ka) [f]	
			Beta dose rate [b,c]	Gamma dose rate [c,d]	Cosmic dose rate [e]	Total dose rate [c,f]	No. of grains [g]	Over-dispersion (%) [h]	Age model [i,j]	D_e (Gy) [f]	
CLO19-2	SU2	13 ± 1	1.12 ± 0.06	0.36 ± 0.01	0.01 ± 0.01	1.53 ± 0.07	152 / 800	88 ± 6	MAM-3	6.2 ± 0.2	4.04 ± 0.27
CLO19-3	SU3A	16 ± 2	1.13 ± 0.06	0.45 ± 0.02	0.01 ± 0.01	1.63 ± 0.08	212 / 1000	112 ± 6	MAM-4	9.1 ± 0.6	5.58 ± 0.47
CLO19-7	SU3C	18 ± 2	1.05 ± 0.05	0.39 ± 0.02	0.01 ± 0.01	1.48 ± 0.07	208 / 1100	96 ± 5	MAM-3	12.8 ± 0.7	8.63 ± 0.66
CLO19-1	SU3D	19 ± 2	1.00 ± 0.05	0.47± 0.02	0.01 ± 0.01	1.51 ± 0.07	202 / 1000	95 ± 5	MAM-3	13.9 ± 0.6	9.22 ± 0.62
CLO19-4	SU3D	20 ± 2	0.89 ± 0.05	0.39 ± 0.02	0.01 ± 0.01	1.33 ± 0.07	182 / 1000	99 ± 6	MAM-3	12.7 ± 0.5	9.50 ± 0.64
CLO19-9	SU3E	21 ± 2	1.12 ± 0.05	0.47 ± 0.02	0.01 ± 0.01	1.63 ± 0.08	154 / 1000	95 ± 6	MAM-4	15.1 ± 1.1	9.27 ± 0.80
CLO19-10	SU3E	25 ± 3	0.96 ± 0.05	0.39 ± 0.02	0.01 ± 0.01	1.39 ± 0.07	205 / 1000	92 ± 5	MAM-4	12.8 ± 0.4	9.22 ± 0.58
CLO19-8	SU3G	24 ± 2	1.02 ± 0.05	0.50 ± 0.02	0.01 ± 0.01	1.57 ± 0.08	188 / 900	96 ± 5	MAM-4	13.4 ± 0.4	8.51 ± 0.53
CLO19-6	SU5A	18 ± 2	0.64 ± 0.03	0.39 ± 0.02	0.01 ± 0.01	1.07 ± 0.05	201 / 1000	37 ± 2	MAM-3	50.3 ± 3.6	46.93 ± 4.15
CLO19-5	SU5B	21 ± 2	0.57 ± 0.03	0.32 ± 0.01	0.01 ± 0.01	0.93 ± 0.05	142 / 700	45 ± 3	MAM-3	48.4 ± 4.4	51.83 ± 5.51

[a] Long-term water content, expressed as % of dry mass of mineral fraction, with an assigned relative uncertainty of ±10%.

[b] Beta dose rates were calculated on dried and powdered sediment samples using a Risø GM-25-5 low-level beta counter†, after making allowance for beta dose attenuation due to grain-size effects and HF etching‡.

[c] Specific activities and radionuclide concentrations have been converted to dose rates using previously established conversion factors§, making allowance for beta-dose attenuation||,‡.

[d] Gamma dose rates were calculated from *in situ* measurements made with a NaI:Tl detector, using the 'energy windows' approach¶.

[e] Cosmic-ray dose rates were calculated# and assigned a relative uncertainty of ±10%.

[f] Mean ± total uncertainty (68% confidence interval), calculated as the quadratic sum of the random and systematic uncertainties.

[g] Number of D_e measurements that passed the SAR rejection criteria and were used for D_e determination / total number of grains analysed.

[h] The relative spread in the D_e dataset beyond that associated with measurement uncertainties, calculated using a central age model☆.

[i] Age model used to calculate the sample-averaged D_e value for each sample. MAM-3 = 3-parameter minimum age model; MAM-4 = 4-parameter minimum age model☆. MAM-3 and MAM-4 D_e estimates have been calculated after adding, in quadrature, a relative error of 20% to each individual D_e measurement error to approximate the underlying dose overdispersion observed in 'ideal' (well-bleached and unmixed) sedimentary samples (e.g., global overdispersion dataset mean value of 20 ± 1%**) and the minimum estimate of intrinsic (experimental) overdispersion determined from the single-grain dose-recovery test for samples CLO19-1 and CLO19-7.

[j] Age model selection: The choice of whether to use the MAM-3 or MAM-4 for each sample has been made on statistical grounds using a maximum log likelihood score (L_{max}) criterion††.

Supplementary References

* David, B. et al. Late survival of megafauna refuted for Cloggs Cave, SE Australia: Implications for the Australian Late Pleistocene megafauna extinction debate. *Quat. Sci. Rev.* (submitted).

† Bøtter-Jensen, L. & Mejdahl, V. Assessment of beta dose-rate using a beta GM multicounter system. *Nucl. Tracks Radiat. Meas.* **14**, 187–191 (1988).

‡ Brennan, B. J. Beta doses to spherical grains. *Radiat. Meas.* **37**, 299–303 (2003).

§ Guérin, G. et al. Dose-rate conversion factors: update. *Ancient TL* **29**, 5–8 (2011).

|| Mejdahl, V. Thermoluminescence dating: beta-dose attenuation in quartz grains. *Archaeometry* **21**, 61–72 (1979).

¶ Arnold, L. J., Duval, M., Falguères, C., Bahain, J.-J. & Demuro, M. Portable gamma spectrometry with cerium-doped lanthanum bromide scintillators: suitability assessments for luminescence and electron spin resonance dating applications. *Radiat. Meas.* **47**, 6–18 (2012).

# Prescott, J. R. & Hutton, J. T. Cosmic ray contributions to dose rates for luminescence and ESR dating: large depths and long-term time variations. *Radiat. Meas.* **23**, 497–500 (1994).

☆ Galbraith, R. F. et al. Optical dating of single and multiple grains of quartz from Jinmium rock shelter, northern Australia: Part I, Experimental design and statistical models. *Archaeometry* **41**, 339–364 (1999).

** Arnold, L. J. & Roberts, R. G. Stochastic modelling of multi-grain equivalent dose (D_e) distributions: implications for OSL dating of sediment mixtures. *Quat. Geochronol.* **4**, 204–230 (2009).

†† Arnold, L. J. et al. A revised burial dose estimation procedure for optical dating of young and modern-age sediments. *Quat. Geochronol.* **4**, 306–325 (2009).
